# Extrahepatic Vascular Parasitization by a Hepatocellular Carcinoma

**DOI:** 10.7759/cureus.5807

**Published:** 2019-09-30

**Authors:** Marco Ertreo, Daniel R Swerdlow, Alexander Kim, Alexander S Somwaru

**Affiliations:** 1 Vascular and Interventional Radiology, Medstar Georgetown University Hospital, Washington, DC, USA; 2 Diagnostic Radiology, Medstar Georgetown University Hospital, Washington, DC, USA; 3 Diagnostic Radiology, Icahn School of Medicine at Mount Sinai, New York, USA

**Keywords:** hepatocellular carcinoma, tumor embolization, microwave ablation, mri, cta, digital subtraction angiography, hcc

## Abstract

Hepatocellular carcinoma is the most common primary hepatic malignancy. For patients not amenable to surgical treatment, transarterial chemoembolization is a viable therapeutic alternative. Extrahepatic collateral arterial supply to the tumor may occur in a variety of scenarios and timely detection of this phenomenon is of fundamental importance to achieve optimal outcomes and response to treatment. This report presents a case of hepatocellular carcinoma that was supplied mainly by a parasitized right phrenic artery and was only successfully treated once this was identified. Further discussion of extrahepatic collateral arterial supply is also presented.

## Introduction

Hepatocellular carcinoma (HCC) is the most common primary hepatic malignancy, the sixth most common cancer overall, and its incidence is expected to increase in the future, particularly in the Western world due to the rising incidence of nonalcoholic steatohepatitis (NASH) [1-2]. It most commonly arises from chronically injured hepatocytes and up to 80% of patients with HCC have underlying cirrhosis [3-4]. HCC is considered a hypervascular tumor deriving its entire vascular supply from hepatic artery branches, and it is not uncommon for it to parasitize adjacent vessels and develop extrahepatic collateral (EHC) arterial supply [5-6]. Timely detection of this phenomenon is of fundamental importance to achieve optimal outcomes and response to transarterial treatments.

## Case presentation

**Figure 1:**
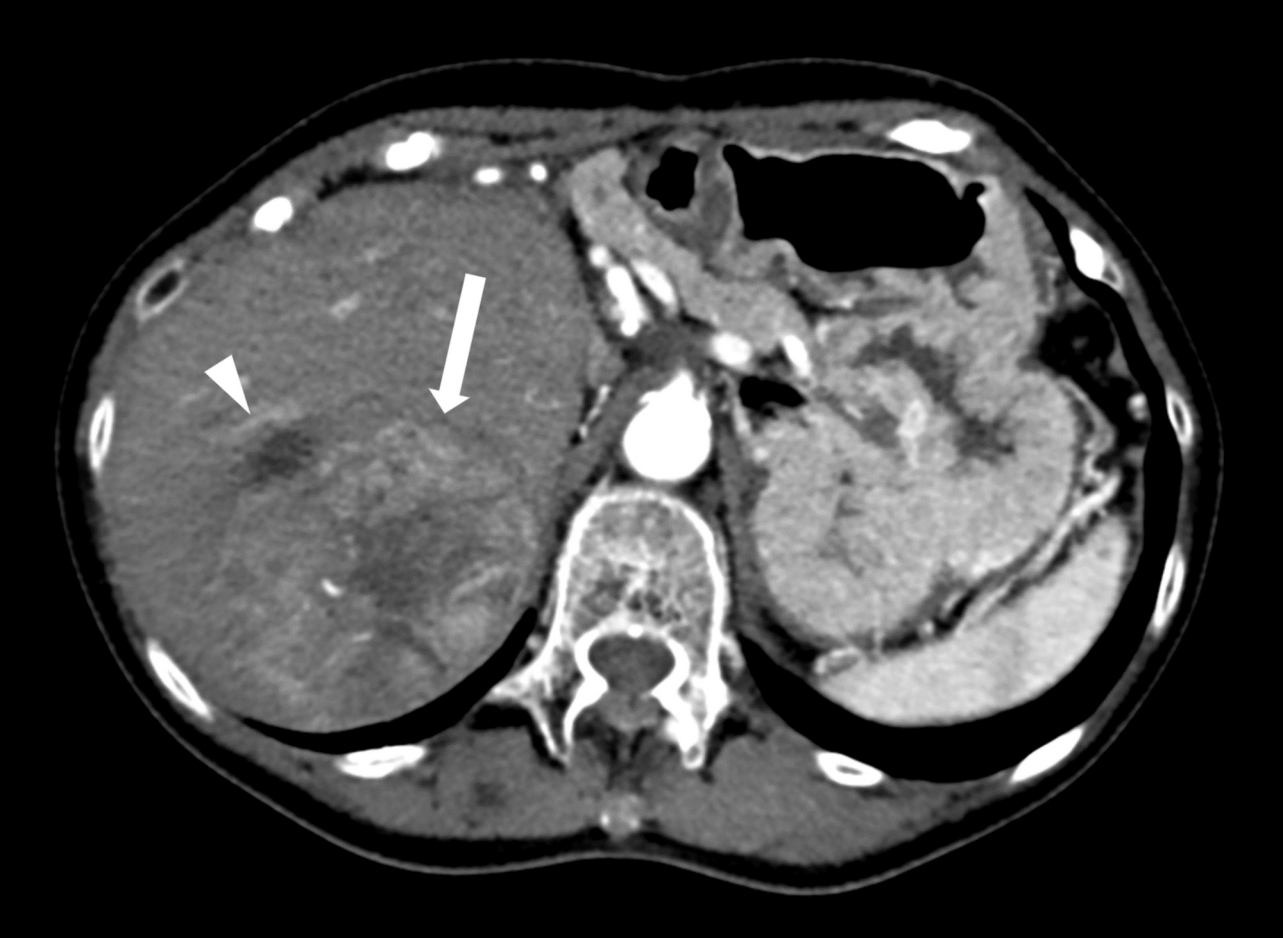
Axial CTA Large, heterogeneously enhancing mass with central necrosis measuring 7.3 x 6.0 cm in the greatest axial dimension (arrow). The lesion is subcapsular and subdiaphragmatic, without extracapsular extension. Partially visualized hypodensity anterior to the lesion is a simple cyst (arrowhead). CTA: computed tomographic angiogram

A 78-year-old woman with a past medical history significant for hepatitis B virus-related cirrhosis was diagnosed with HCC in segments 2 and 3 and initially treated with surgical resection of the left lateral segment. On follow-up imaging 18 months later, she was found to have large, local tumor recurrence in segment VII and was subsequently referred to the Interventional Radiology clinic for liver-directed therapy (Figure 1).

**Figure 2:**
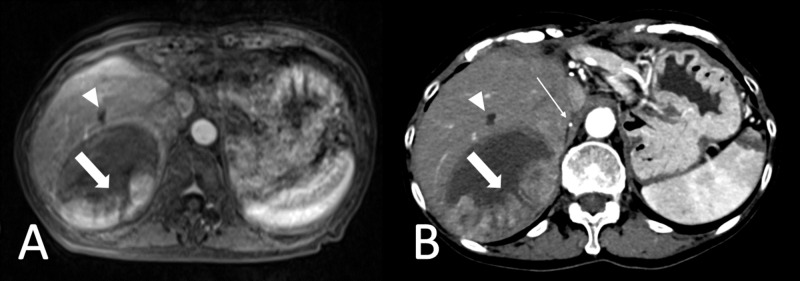
A) Axial MR image, T1-weighted 3D gradient-echo, delayed arterial phase. Status post TACE with a large embolization cavity and persistent peripheral enhancing nodular components (arrow). Partially visualized anterior to the embolization cavity is a simple cyst. B) Axial CTA image. Status post TACE with large embolization cavity with persistent peripheral enhancing nodular components (thick arrow) and adjacent hypertrophied right phrenic artery (thin arrow). Partially visualized hypodensity anterior to the lesion is a simple cyst. MR: magnetic resonance; 3D: three-dimensional; TACE: transarterial chemoembolization; CTA: computed tomographic angiogram

**Figure 3:**
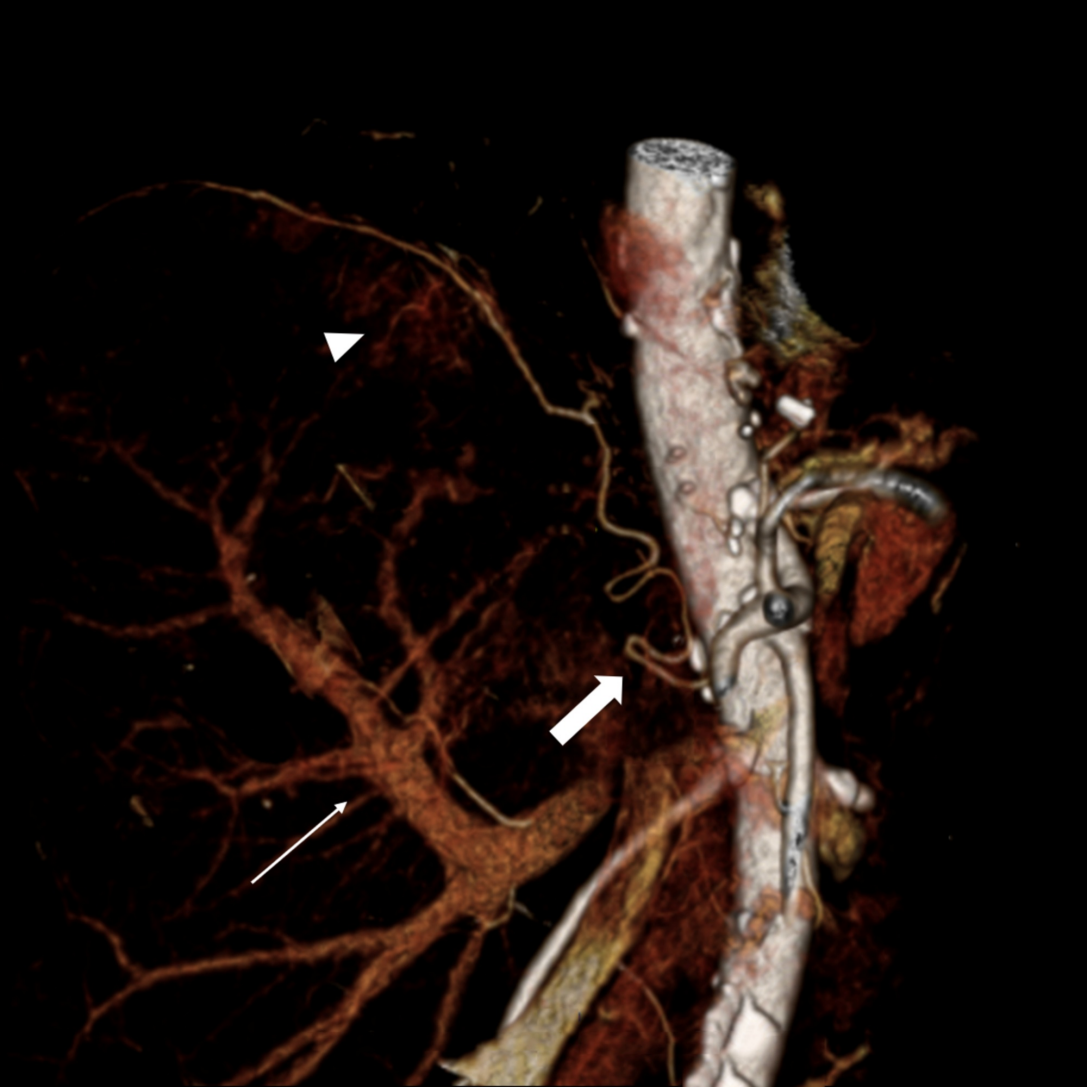
Computed tomography, 3D reconstruction Hypertrophied and parasitized right phrenic artery (thick arrow) providing extra-hepatic collateral supply to the subdiaphragmatic enhancing nodular component within the embolization cavity (arrowhead). Early enhancement of the portal venous system is also seen (thin arrow). 3D: three-dimensional

She underwent transarterial chemoembolization (TACE) of the right hepatic artery branches, although follow-up magnetic resonance imaging (MRI) demonstrated persistent tumor viability, as evidenced by nodular enhancement within the embolization cavity, located peripherally adjacent to the diaphragm (Figure 2A). Given these findings, she underwent a second TACE procedure in a similar fashion. Follow-up imaging once again demonstrated a viable tumor in the embolization cavity and, additionally, serum alpha-fetoprotein levels continued to rise. A computed tomographic angiogram (CTA) of the abdomen was obtained to further delineate the vascular supply to the tumor. This demonstrated EHC arterial supply arising from a hypertrophied right inferior phrenic artery and several first and second division branches (Figures 2B-3).

**Figure 4:**
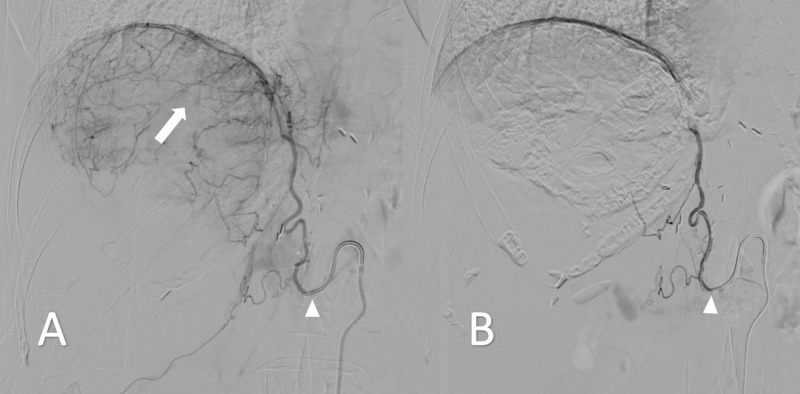
Intraprocedural digital subtraction angiogram A) Proximal right phrenic angiogram with the catheter tip at the vessel origin (arrowhead), demonstrating peripheral tumoral enhancement (arrow), consistent with known residual disease. B) Post-embolization proximal right phrenic angiogram with catheter tip at the vessel origin (arrowhead), demonstrating a lack of subdiaphragmatic nodular enhancement.

Once this vessel was identified, the patient underwent TACE via the nutrient vessel (Figure 4). This was performed selectively catheterizing the right phrenic artery, confirming supply to the hepatic tumor (Figure 4A), and then injecting doxorubicin loaded onto microspheres, followed by bland embolization. Repeat arteriogram following embolization demonstrated absent arterial supply to the tumor (Figure 4B).

**Figure 5:**
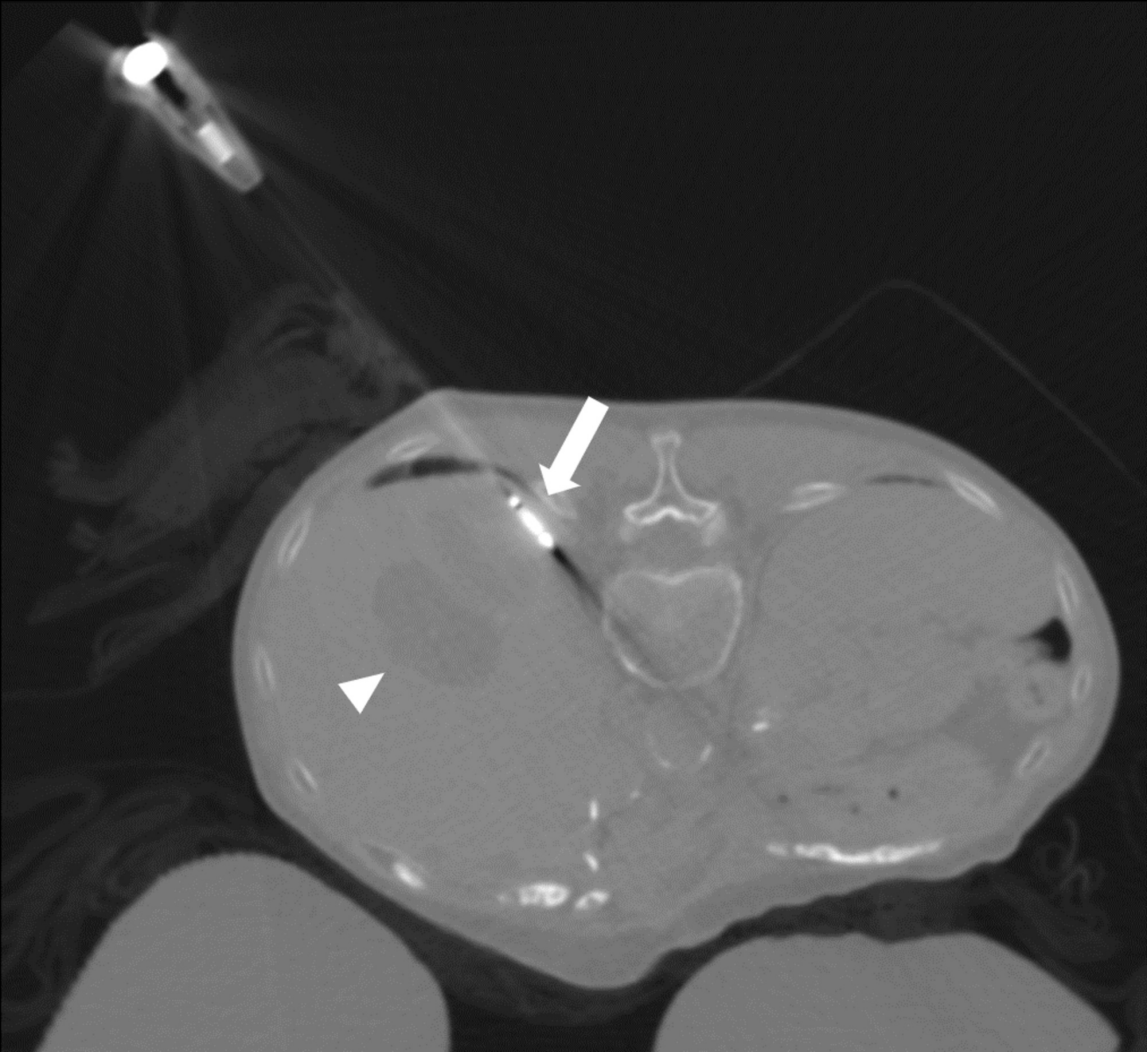
Intraprocedural ablation axial CT image Microwave ablation antenna positioned in the subdiaphragmatic nodular component of the embolization cavity (arrow). Anterior to this is a partially visualized simple cyst (arrowhead). CT: computed tomography

The patient subsequently underwent CT-guided microwave ablation of the lesion (Figure 5). This was performed with the patient in the prone position and under general anesthesia. A microwave antenna was advanced into the subdiaphragmatic nodular component under CT guidance and microwave ablation was performed. The needle was then withdrawn without complications.

**Figure 6:**
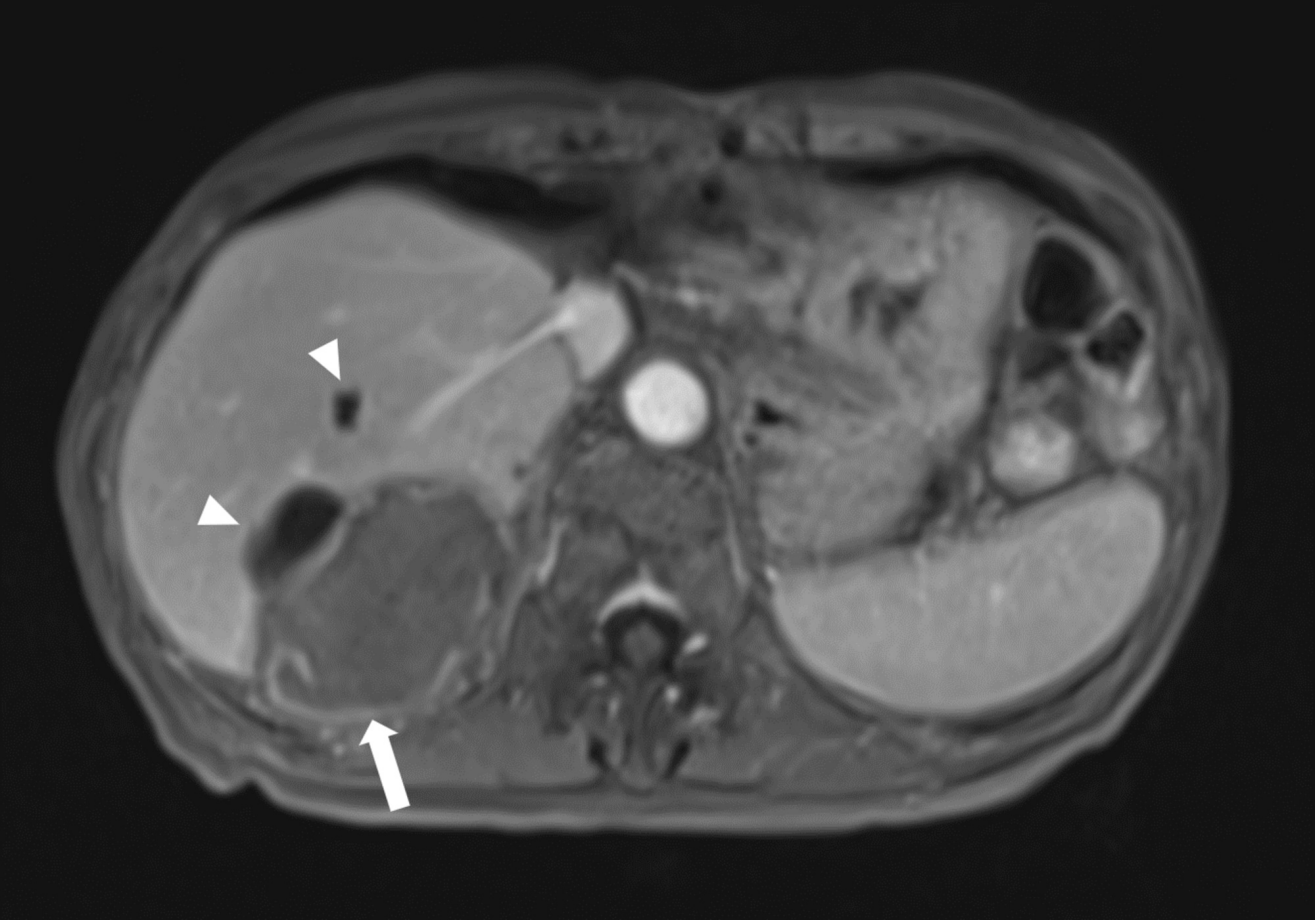
Axial MR image, T1-weighted 3D gradient-echo, late arterial phase Post-treatment ablation cavity without enhancing nodules or masses (arrow). Rim enhancement of the cavity is consistent with granulation tissue. Partially visualized anterior to the embolization cavity are two simple cysts (arrowheads). MR: magnetic resonance; 3D: three-dimensional

Follow-up imaging demonstrated a complete response with a lack of any internal enhancement (Figure 6).

## Discussion

HCC is a hypervascular tumor typically deriving its blood supply from hepatic artery branches. At any point during its lifespan, it may parasitize adjacent vessels and develop EHC arterial supply [5-7]. Timely detection of this phenomenon is of fundamental importance to achieve optimal outcomes and response to treatment, as seen in the case presented above. Certain conditions predispose to parasitization and should be kept under consideration when reviewing imaging or treatment planning. Established risk factors predisposing to EHC formation include large tumors (>5 cm), exophytic growth with extrahepatic infiltration, peripherally growing tumors or those near the bare area of the liver, prior surgery, or transarterial interventions, such as TACE, with injury to the hepatic artery [6-8]. Additionally, EHC supply should be suspected when peripheral nodular enhancement persists opposite to the main hepatic arterial supply inflow and despite multiple TACE procedures (as in this case), when there is continued elevation of serum alpha-fetoprotein levels or when part of the lesion demonstrates greater enhancement or lipiodol accumulation (if used during TACE) [5-6]. The most common vessel to give rise to EHC supply is the right inferior phrenic artery (IPA), with prevalence ranging between 38% and 83% of cases [5-7,9]. Other vessels that may be parasitized include the right or left internal mammary, right adrenal, cystic, renal, gastric, or omental arteries, with different rates reported in the literature [6,9]. In the case presented, EHC supply arose from a hypertrophied right IPA and several first and second divisional branches. Typically, the IPA arises from either the abdominal aorta or the celiac arterial trunk (less frequently from direct branches of the aorta such as the renal arteries) and courses superiorly along the bare surface of the liver to supply the ipsilateral hemidiaphragm, anastomosing with terminal branches of the internal mammary and/or intercostal arteries. Diagnostic and interventional radiologists should suspect EHC supply when a tumor arises in hepatic segment VII or is in contact with the right hemidiaphragm, which may mandate the performance of selective angiography of the right IPA at the time of treatment [5-6,9]. Similarly, a tumor abutting the left hemidiaphragm or arising in the left lobe (segments II and III) warrants vascular interrogation of the left IPA [9].

Treatment for HCC varies greatly based on tumor staging and underlying remaining liver function [10-12]. Many staging systems are available to guide treatment but the only system that takes into consideration tumor staging, underlying liver dysfunction, and the patient’s functional status is the Barcelona Clinic Liver Cancer (BCLC) classification [10-12]. Ultimately, given the complexity and heterogeneity of the patient population, there is often a multidisciplinary evaluation in order to determine the optimal treatment for each. The patient presented above was classified as BCLC B (intermediate disease with good functional status and preserved liver function). Consequently, she underwent TACE followed by percutaneous microwave ablation (MWA). This combined approach (TACE followed by ablation) is being increasingly used, with data demonstrating improved overall survival and prognosis compared to monotherapy with TACE alone, particularly for lesions smaller than 3 cm [13-15]. TACE-MWA combination therapy is thought to be synergistic, as embolization delivers in situ chemotherapeutic drugs and reduces arterial blood flow to the lesion, therefore, reducing blood flow-mediated heat loss, known as “heat sink,” and one of the main drawbacks of percutaneous ablative therapies, mainly radiofrequency mediated [13,16]. Following the combined treatments, the lesion underwent a complete response with a lack of any nodular enhancement on follow-up imaging.

## Conclusions

TACE is accepted therapy for intermediate-stage HCC. The possibility of EHC supply to a lesion should be considered and investigated when predisposing risk factors are present or imaging findings and clinical course are suggestive of alternative vascular supply. Referring clinicians and radiologists, both diagnostic and interventional alike, must be aware of this phenomenon. Early detection is fundamental for timely and successful treatment.
